# The potential for measles transmission in England

**DOI:** 10.1186/1471-2458-8-338

**Published:** 2008-09-26

**Authors:** Yoon Hong Choi, Nigel Gay, Graham Fraser, Mary Ramsay

**Affiliations:** 1 Centre for Infections, Health Protection Agency, Colindale, NW9 5EQ, London; 2 Health Protection Agency, WC1V 7PP, London

## Abstract

**Background:**

Since the schools vaccination campaign in 1994, measles has been eliminated from England. Maintaining elimination requires low susceptibility levels to keep the effective reproduction number R below 1. Since 1995, however, MMR coverage in two year old children has decreased by more than 10%.

**Methods:**

Quarterly MMR coverage data for children aged two and five years resident in each district health authority in England were used to estimate susceptibility to measles by age. The effective reproduction numbers for each district and strategic health authority were calculated and possible outbreak sizes estimated.

**Results:**

In 2004/05, about 1.9 million school children and 300,000 pre-school children were recorded as incompletely vaccinated against measles in England, including more than 800,000 children completely unvaccinated. Based on this, approximately 1.3 million children aged 2–17 years were susceptible to measles. In 14 of the 99 districts, the level of susceptibility is sufficiently high for R to exceed 1, indicating the potential for sustained measles transmission. Eleven of these districts are in London. Our model suggests that the potential exists for an outbreak of up to 100,000 cases. These results are sensitive to the accuracy of reported vaccination coverage data.

**Conclusion:**

Our analysis identified several districts with the potential for sustaining measles transmission. Many London areas remain at high risk even allowing for considerable under-reporting of coverage. Primary care trusts should ensure that accurate systems are in place to identify unimmunised children and to offer catch-up immunisation for those not up to date for MMR.

## Background

Measles vaccination was introduced in the UK in 1968 for children in the second year of life [1]. Coverage gradually improved from approximately 50% during the 1970s to 86% when MMR vaccine replaced single antigen vaccine in 1988, and reached 92% in 1995. As a result, measles epidemics, which had occurred biennially in the pre-vaccination period with hundreds of thousands of notified cases, became smaller and less frequent. Control of measles reached a new level following a national vaccination campaign in November 1994, when measles-rubella vaccine was offered to all school children aged 5–16 years to prevent a predicted epidemic of measles; coverage of 92% was achieved and endemic transmission of measles was interrupted [2]. In 1996, a second dose of MMR was added to the routine vaccination schedule at around 4 years of age. Despite the occurrence of occasional outbreaks, caused by limited secondary spread from imported cases, measles has been eliminated in England and Wales for more than 10 years [3].

To maintain measles elimination, the effective reproduction number, R, (the average number of secondary cases infected by a typical infectious case) needs to remain below 1 [4]. This can be achieved through maintaining low levels of susceptibility by vaccinating a high proportion of the population. The European region of the World Health Organisation (WHO) defined target levels of susceptibility to sustain measles elimination: the level of susceptibility in 1–4 year olds should be less than 15%, in 5–9 year olds less than 10% and in all older cohorts less than 5% [5]. As around 90% of individuals are protected from measles after a first dose of vaccine, and 99% after a second dose, to achieve these target levels requires high vaccination coverage to be sustained [6].

Coverage for MMR vaccine in two year old children in England fell from the peak of 92% in 1995 to reach the low of 80% in 2003. Coverage in London is lower than in the rest of England. We have used routine data on vaccine coverage in two and five year old children to estimate the level of susceptibility, and hence the reproduction number for measles in England. These results can be used to predict future control of measles and to estimate the possible impact of measles becoming re-established [7].

## Methods

### Vaccination Coverage

Between 1995 and 2002, quarterly and annual data on coverage of MMR in two and five year old children, collected as part of the COVER (Cover of Vaccination Evaluated Rapidly) programme, was available by former district health authorities (DHA) [8]. Since the reorganisation of the NHS in 2002, data for England is collected from primary care trusts (PCTs). To provide consistent data for all childhood cohorts, coverage from each PCT was aggregated to the district health authority (DHA) configuration prior to 2002 and to the Strategic Health Authority (SHA) configuration as over the period 2002–2006.

At age two years the denominator and the number of these children who have received one dose of MMR are reported; at age five years the denominator, the number of these children who have received at least one dose of MMR, and the number who have received two doses of MMR are reported. This enables the proportion of the cohort who have received no doses, one dose only and two or more doses to be calculated.

Three procedures were adopted to deal with missing and anomalous coverage data.

1) Interpolation was performed to eliminate inconsistent and missing data for any one quarter. When data for a single quarter was missing, the data was assumed to be the same as the previous quarter. Where data for more than one successive quarter was not available, linear interpolation from the last available quarter to the next available quarter was used.

2) The coverage of MMR in children born in 1990 and 1991 was not captured by routine data at five years of age, so coverage of the first dose at this age was assumed to be the same as for children born after March 1992 (available from COVER). Children born between January 1990 and March 1992 were neither in the target age group for the national MR catch-up campaign in 1994 nor eligible for routine MMR2, but were scheduled to receive a second dose in a catch-up in October 1996. Coverage in this group varied from 47.6% in South Thames region to 66.8% in Northern and Yorkshire and so it was assumed that 50% of these cohorts received the second dose.

3) Data at age two years is believed to be reasonably accurate, but coverage at age five years is thought to under-estimate the true coverage in many trusts [9]. This may be caused by children who have left the area not being removed from the denominator or by incomplete recording of vaccination history for children who move into the area. For example, eight DHAs reported a lower vaccination coverage for one dose at five years than for the same cohort at two years of age. To correct this problem, coverage at five years of age was corrected to be at least 3% higher than the two year old figure in each district. This increase in coverage was the mean increase observed between two and three years of age in sentinel trusts.

In addition, a sensitivity analysis was conducted to estimate the potential impact of under-estimating coverage. An audit of data quality for children born between July and September 1995 in 12 London PCTs in 2001 suggested that around 24% (201/836) of children recorded as unvaccinated for MMR at five years of age had received at least one dose of vaccine. Therefore, our analysis was repeated assuming that 10%, 20%, 30%, 40% and 50% of children recorded as unvaccinated had received one dose of vaccine and that 10%, 20%, 30%, 40% and 50% of those who were recorded as receiving a single dose had also received the second dose.

### Susceptibility

The relevant quarterly birth cohorts were aggregated to approximate each school year (born September–August) and vaccine coverage for MMR1 and MMR2 calculated. For example, children born between October 1999 and September 2000 comprised the four year old cohort in the school year 2004/2005. Susceptibility was then estimated for the pre-school group (aged 0–4 years), infant and junior school (aged 5–10 years), secondary school (aged 11–17 years), college (18–24 years) and older (25 years or more) [10].

Cohorts born since 1990 have almost no exposure to natural measles infection, so the proportion susceptible in each age cohort is calculated as follows:

$$\begin{array}{ccc} \text{Proportion susceptible}\; & = & \text{1}00\%\times \text{ Proportion with }0\text{ doses}\\ & & +\text{ 1}0\%\;\times \text{ Proportion with 1 dose only}\\ & & +\text{ 1}\%\;\times \text{ Proportion with 2 doses} \end{array}$$

Children are assumed to be immune for the first six months of life through maternal antibody. For cohorts born before 1990, susceptibility was assumed to be 5% for secondary school and college (14–24 years) and 2% for older (25 years+) based upon sero-prevalence studies conducted after the national vaccination campaign [11,12].

To calculate the susceptibility to measles in years after 2004/2005 it was assumed that vaccination coverage remained stable at 2004/2005 levels. This assumption was necessary for two reasons. First, coverage data from April–June 2005 to date has been incomplete due to problems with the implementation of new child health computer systems in several London PCTs [13]. Second, from October 2006 reconfiguration of the number of PCTs in England from 303 to 152, means that mapping these data to old DHA areas would be more complex [14].

### Effective Reproduction Number

The effective reproduction number (R) for each previous DHA and SHA was calculated from the Next Generation Matrix (NGM) accounting for the proportion susceptible in each age group [11,15]. This matrix accounts for age-specific heterogeneity in measles transmission. The values for a totally susceptible population are shown below, which give an R_0 _of 10.7.

$$NGM=\left(\begin{array}{ccccc} \text{1}\text{.92} & \text{0}\text{.38} & \text{0}\text{.38} & \text{0}\text{.38} & \text{0}\text{.38}\\ \text{0}\text{.47} & \text{4}\text{.98} & \text{1}\text{.80} & \text{0}\text{.47} & 0.47\\ \text{0}\text{.38} & \text{1}\text{.80} & 7.48 & \text{0}\text{.47} & 0.47\\ 0.47 & 0.47 & 0.47 & 8.72 & 0.47\\ 5.22 & 5.22 & 5.22 & 5.22 & 5.22 \end{array}\right)$$

For areas where R exceeds one, the potential outbreak size was estimated as twice the number of infections required to reduce R to 1 in that area [3,16].

## Results

**Figure 1:**
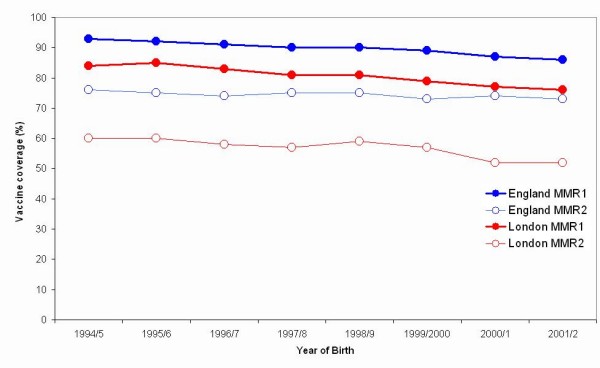
**Reported MMR vaccination coverage at five years of age in England and London, 1992–2004.** (MMR1 for children who received at least 1 dose and MMR2 for children who received two doses).

Coverage of English children born between September 1992 and August 2002 by five years of age ranged from 89.5% to 94.7% for at least one dose of MMR and from 73.7% to 75.0% for two doses (Figure 1). Coverage of MMR in London was lower than in England overall by 7% (for one dose) and 16% (for two doses) (Figure 1). Coverage at five years declined over time, by 5.3% for MMR1 and 0.8% for MMR2

**Figure 2:**
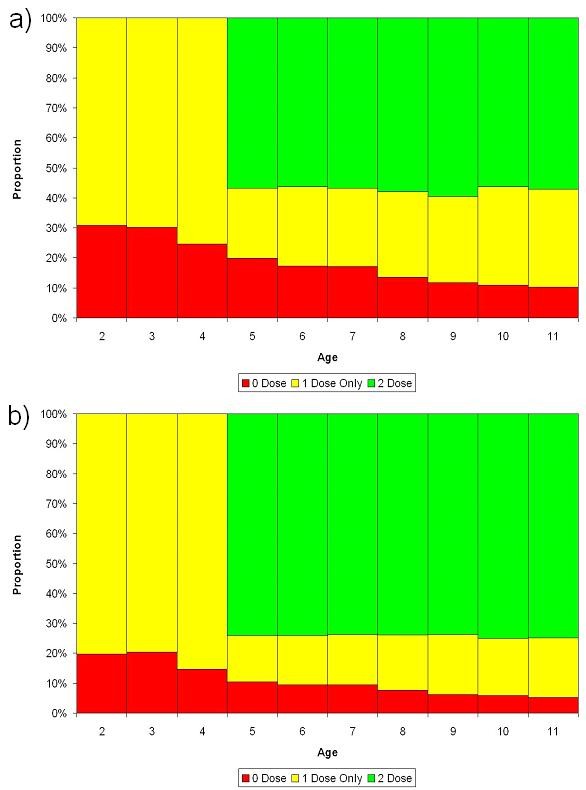
Derived vaccination status by age, 2004/5: a) England, b) London.

Figure 2 displays the estimated vaccination status for each birth cohort in England. The proportion unvaccinated has risen from 5.1% in the 11 year old cohort to 10% in the 5 year cohort.

Table 1 presents the estimated MMR vaccination status of children in each school age group using cleaned routine data. In all 1.9 million school children (5–17 years) and 0.3 million pre-school children are incompletely vaccinated for their age (i.e. have not received the scheduled doses). Of these, more than 800,000 children are completely unvaccinated. The number of susceptible children between 2 and 17 years was estimated to be 1.1 million in 2004/2005 (Table 1). Using our highest estimate of misclassification, if 50% of children recorded as unvaccinated were actually partially vaccinated and the same proportion of partially vaccinated children actually fully vaccinated, the revised numbers would be 1.6 million incompletely vaccinated children, (of whom 0.4 million completely unvaccinated) leading to 0.7 million susceptible children.

**Table 1:** Numbers of children (000s) by vaccination status and number of susceptibles by age group in England in 2004.

**School cohort (approximate age group)**	**Total**	**MMR vaccine received**			**Susceptibles n (%)**
		**None**	**One dose**	**Two doses**	
**Pre-school (2–4 years old)**	1663	320	1343	0	454 (27%)
**Primary school (5–10 years old)**	3582	271	627	2684	360 (13%)
**Secondary school (11–17 years old)**	3656	211	826	2619	320 (9%)

### Susceptibility by birth cohort

**Figure 3:**
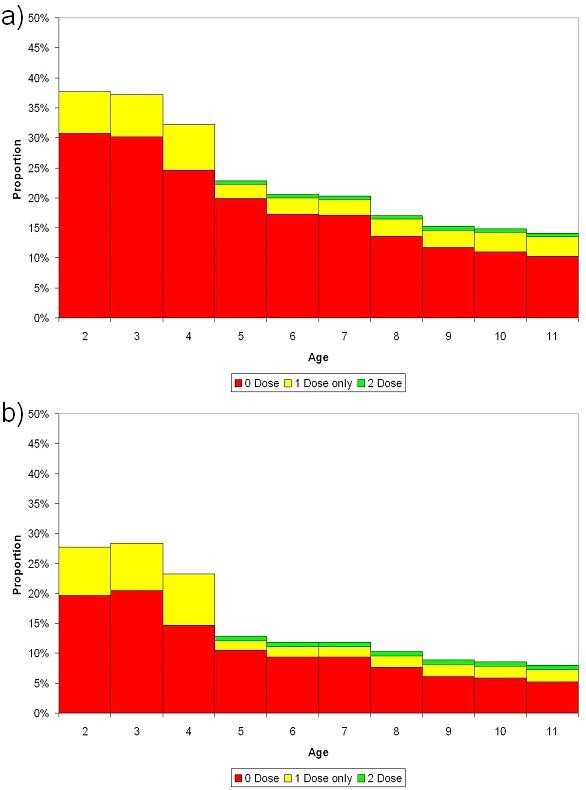
Calculated proportion susceptible to measles, 2004/5 by age and vaccination status: a) England, b) London.

Figure 3 shows the susceptibility of each school year cohort in England (all districts combined) calculated from the cleaned routine data and using estimates of vaccine efficacy. The overall proportion susceptible was 27% among 2–4 year olds (born 2000–2002), 13% among children among primary school children (5–10 year olds) and 9% among secondary school children. However there was considerable variation between districts ranging from 19% – 46% and 4% – 27% in 2–4 and 5–10 year olds respectively. The majority of susceptible children are completely unvaccinated with MMR: of susceptible primary school children, 74% have received 0 doses, 23% a single dose, and 3% two doses.

### Calculated values of *R*

The number of DHAs in 28 SHAs in four different R bands are shown in Table 2. In 14 districts the levels of susceptibility were sufficiently high for R to exceed 1, indicating the potential for sustained measles transmission. Eleven of these 14 DHAs are located in London, with only 5 DHAs in London having R below 1. In a further seven DHAs, R was close to the threshold in the range 0.90–0.99, 10 DHAs in the range 0.80–0.89, and 68 DHAs had R values lower than 0.8.

**Table 2:** Number of District Health Authorities (DHAs) in different R bands in 28 Strategic Health Authorities (SHAs) in 2004/05

SHA name	>= 1	0.9 – 0.99	0.8 – 0.89	<0.8
NORFOLK, SUFFOLK AND CAMBRIDGESHIRE HA				3
BEDFORDSHIRE AND HERTFORDSHIRE HA			1	2
ESSEX HA				2
NORTH WEST LONDON HA	3		1	
NORTH CENTRAL LONDON HA	1	2		
NORTH EAST LONDON HA	1			2
SOUTH EAST LONDON HA	3			
SOUTH WEST LONDON HA	3			
NORTHUMBERLAND, TYNE & WEAR HA				4
COUNTY DURHAM AND TEES VALLEY HA			1	1
NORTH AND EAST YORKSHIRE AND NORTHERN LINCOLNSHIRE				3
WEST YORKSHIRE HA			2	2
CUMBRIA AND LANCASHIRE HA				5
GREATER MANCHESTER HA		1		4
CHESHIRE & MERSEYSIDE HA		1	2	3
THAMES VALLEY HA	1			2
HAMPSHIRE AND ISLE OF WIGHT HA			1	3
KENT AND MEDWAY HA				2
SURREY AND SUSSEX HA	1	1	1	1
AVON, GLOUCESTERSHIRE AND WILTSHIRE HA				4
SOUTH WEST PENINSULA HA				3
DORSET AND SOMERSET HA				1
SOUTH YORKSHIRE HA		1		3
TRENT HA	1	1		4
LEICESTERSHIRE, NORTHAMPTONSHIRE AND RUTLAND HA				2
SHROPSHIRE AND STAFFORDSHIRE HA				3
BIRMINGHAM AND THE BLACK COUNTRY HA			1	5
COVENTRY, WARWICKSHIRE, HEREFORDSHIRE AND WORCESTERSHIRE				4
Total	14	7	10	68

**Figure 4:**
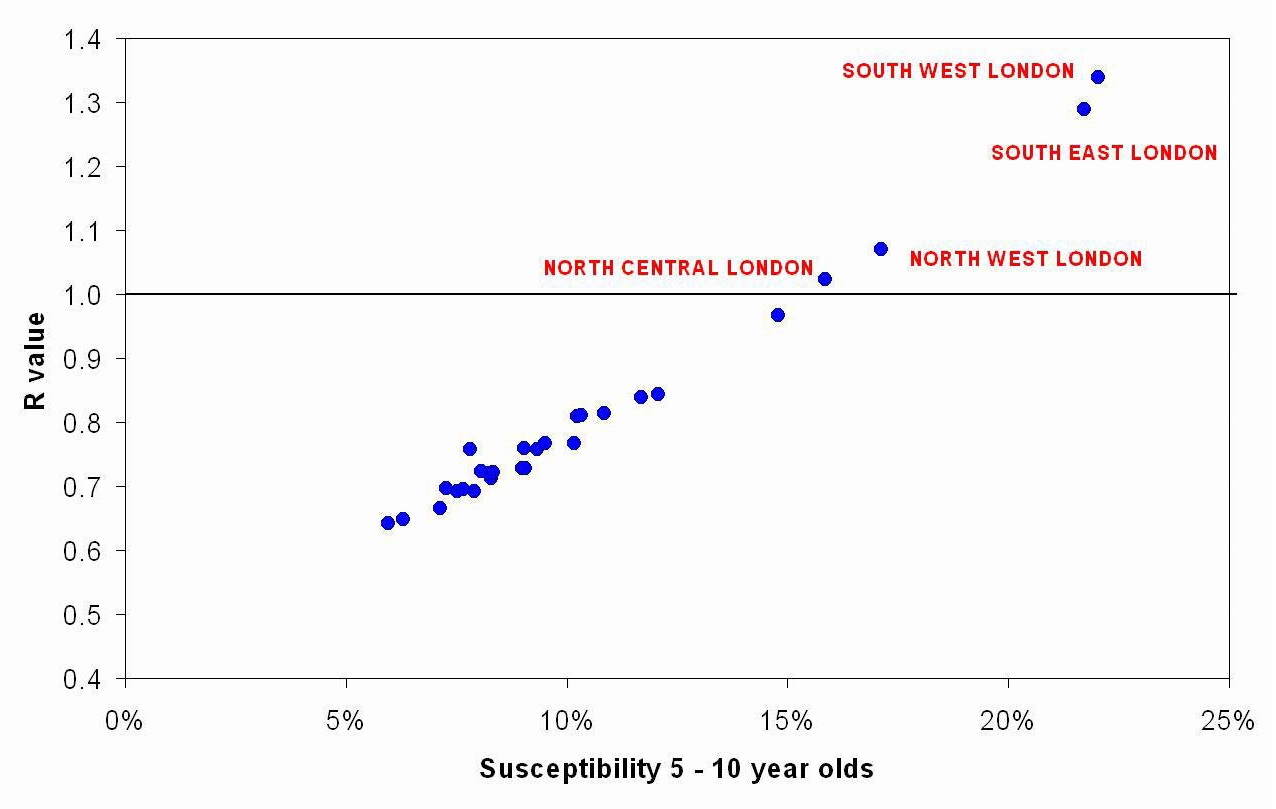
R values in 28 Strategic Health Authorities in England, 2004/05.

When the data are analysed by SHA rather than DHA, only four SHAs (all in London) appear at risk of sustained measles transmission (Figure 4).

### Future control

**Figure 5:**
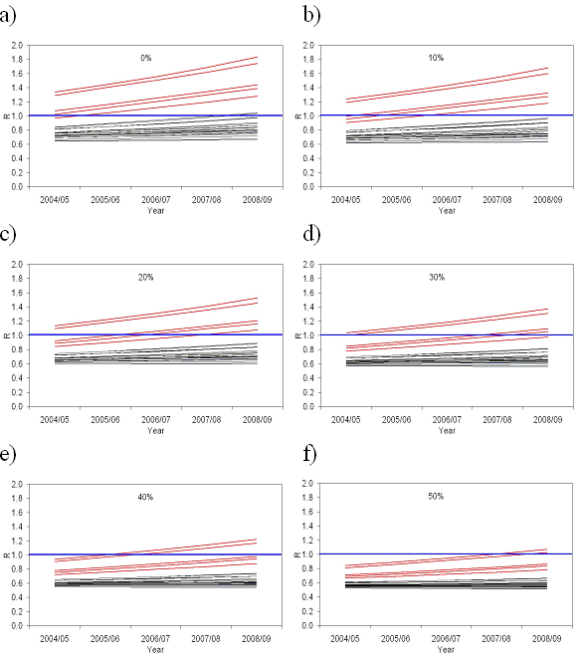
**(a-f).** Evolution of the effective reproduction number, R, from 2004–05 to 2008–09 in the 28 Strategic Health Authorities in England for six possible scenarios for the under-estimation of vaccination coverage (the five SHAs in London are shown in red); the proportion of children recorded as unvaccinated who had received one dose and the proportion of children recorded as having received a single dose who had received two doses was assumed to be : a) 0%, b) 10%, c) 20%, d) 30%, e) 40% and f) 50%.

If coverage remained stable after 2004/05, the total number of susceptible children aged 2–17 years would increase to around 1.2 million by 2007/8. After this time the entire school population would comprise cohorts not covered by the 1994 national vaccination campaign. Thus in SHAs that did not achieve higher coverage, the increase in susceptibility would further increase the value of R (Figure 5).

If the COVER data give a true indication of the vaccination coverage in London, (0% under-reporting, Figure 5a) then R has exceeded 1 in all five London SHAs since 2004/5, and would be as high as 1.34 in South East London. In these circumstances, it is surprising that no major epidemic of measles has already occurred, given the frequent introductions of imported cases. This therefore supports the belief that COVER data underestimates measles vaccination coverage in London, but the degree is uncertain. However, unless at least 50% of those reported as unvaccinated have received measles containing vaccine, the potential for an epidemic in one or more SHAs in London is reached by 2007/8 (Figure 5).

### Outbreak size

The potential outbreak sizes are also sensitive to under-estimation of vaccination coverage. The total potential sizes for the DHAs where R exceeds 1 and are shown in Table 3.

**Table 3:** The potential measles outbreak size (000s) by age group, under four scenarios of under-estimation of vaccine coverage.

Age group	Assumed level of under-estimation in vaccine coverage*			
	20%	30%	40%	50%
0–4 years	22.7	13.0	5.9	1.3
5–10 years	49.9	26.8	11.3	2.2
11–17 years	39.3	22.3	9.5	1.8
18–24 years	3.3	2.0	0.9	0.2
25 years+	9.9	5.8	2.7	0.6
Overall	125.2	70.1	30.6	6.3
In London	105.8	61.8	29.6	6.3

* The proportion of children recorded as unvaccinated who had received one dose and the proportion of children recorded as having received a single dose who had received two doses was assumed to be 20%, 30%, 40% or 50%.

Depending on the degree of under-estimation of vaccination coverage assumed, the model suggests that in 2007/8 the potential exists for a measles outbreak of up to approximately 100,000 cases. Most of these cases would be in school age children in London (Table 3).

## Discussion

Our analysis has identified several DHAs and SHAs in England with the potential for sustaining measles transmission. Among 99 former DHAs, fourteen have R values above 1, meaning that they run the risk of re-establishment of measles transmission in the community. Such transmission could lead to an outbreak involving many thousands of measles cases. A measles epidemic in Italy in 2002 led to around 40,000 cases, with direct costs estimated at between €9.9 and €12.4 million, and total costs around €14.8 million [17]. With such information, the outbreak sizes estimated in this paper can be a valuable tool to estimate the epidemic costs and the cost effectiveness of possible interventions in England, such as a catch-up campaign.

Our analysis ignores immunity through natural infection in children aged under 17 years, as the incidence of measles has been too low to have a significant effect in the cohorts considered. The last national epidemic was in 1988 and the incidence declined further following the national vaccination campaign in November 1994 [2]. The quality of the COVER data is a weakness of this study, as it is likely to underestimate the true vaccine coverage, as some vaccinations are not be recorded on the child health computer system and the total number of children (the denominator) may be inaccurate [9,18]. There has been no published validation of data held for children aged five years, but it is expected that the quality of coverage data would decline with the age of the child as movements out of the denominator are often not recorded. Data is less accurate in populations of high mobility, and therefore the accuracy of COVER data for London is likely to be worse than for the rest of country. Outside of London, some of the DHAs with R above the epidemic threshold are known to have problems with data accuracy. Our estimates of susceptibility to measles by age in England are consistent with data from serological surveillance [12]. Unfortunately few samples in these serosurveys are collected in London, so these data do not reduce the uncertainty regarding vaccination status and susceptibility in London. Even after allowing for considerable under-estimation of coverage, however, many London areas still remain at high risk. In addition, unless routine coverage can be further improved and sustained, the total number of susceptibles will be expected to increase each year. Although there has been an increase in MMR1 coverage at two years since 2003, this is offset by a small decline at five years, suggesting that further accumulation of susceptibles will lead to a higher risk of measles outbreaks in the future [19].

In response to earlier analyses of measles risk for London DHAs, primary care trusts across the city conducted a catch-up campaign among primary school aged children to reduce the number of susceptibles in London [6]. To eliminate the risk of a major outbreak, the campaign would have had to vaccinate 40% of unvaccinated primary school children. More than 40,000 children in this age group were identified and vaccinated by the campaign, including over 17,000 previously unvaccinated children. Initial evaluation indicates, however, that the campaign has achieved only a modest reduction to the epidemic risk in London and that endemic measles transmission risk remains high in several health districts [20].

Each health area should review the vaccination status of school children born since 1990 in order to validate the coverage data, and if necessary, to focus on reducing the proportion of children who are completely unvaccinated against measles. The threat of re-establishing measles transmission could be removed if the proportion of each cohort susceptible to measles was reduced to less than 10% in primary school and to less than 5% in secondary school. Opportunities to improve routine coverage need to be reinforced and supplementary vaccination initiatives for children already attending school will also have to be taken in some areas. Catching-up children born between 1997 and 2003, the cohorts most affected by the decline in MMR coverage should be a priority. A catch-up programme of this type has recently been announced by the Chief Medical Officer [21]. Later cohorts can then be assessed at school entry on a routine basis. A further opportunity at secondary school entry, when a higher level of immunity is required to prevent transmission, should be formalised in each health area. To reduce the number of young adults leaving school susceptible to measles, mumps or rubella, MMR vaccine should also be offered to all unvaccinated or partially vaccinated adolescents at the time of the school leaving booster. If this final opportunity is not taken, adults will be difficult to access in the event of an increase in incidence. This problem has been graphically illustrated by epidemics of rubella in 1996/7 and mumps in 2004/5 [3,4].

## Conclusion

We identified several areas with the potential for sustaining measles transmission. A number of former London health districts remained at high risk of a measles epidemic even allowing for considerable under-reporting of vaccination coverage. Most of those susceptible to measles are unvaccinated children under 17 years old. Vaccinating a significant proportion of these children in the highest risk areas is necessary to remove the risk of an epidemic. Primary care trusts should ensure that accurate systems are in place to identify unimmunised children and to offer catch-up immunisation for those not up to date for MMR.

## Competing interests

The authors declare that they have no competing interests.

## Authors' contributions

YHC cleaned the COVER data, programmed the model in MS VB6.0, analysed the results, and prepared the manuscript. MR aggregated the MMR COVER data for the study and helped writing the manuscript. NG helped with the analysis and writing the manuscript. GF proposed the London analyses in connection with the immunisation catch-up programme and helped writing the manuscript.

## Pre-publication history

The pre-publication history for this paper can be accessed here:


